# Repertoire comparison of the B-cell receptor-encoding loci in humans and rhesus macaques by next-generation sequencing

**DOI:** 10.1038/cti.2016.42

**Published:** 2016-07-22

**Authors:** Vladimir Vigdorovich, Brian G Oliver, Sara Carbonetti, Nicholas Dambrauskas, Miles D Lange, Christina Yacoob, Will Leahy, Jonathan Callahan, Leonidas Stamatatos, D Noah Sather

**Affiliations:** 1 Center for Infectious Disease Research (formerly Seattle BioMed), Seattle, WA, USA; 2 Fred Hutchinson Cancer Research Center, Viral and Infectious Disease Division, Seattle, WA, USA; 3 Mazama Science, Seattle, WA, USA

## Abstract

Rhesus macaques (RMs) are a widely used model system for the study of vaccines, infectious diseases and microbial pathogenesis. Their value as a model lies in their close evolutionary relationship to humans, which, in theory, allows them to serve as a close approximation of the human immune system. However, despite their prominence as a human surrogate model system, many aspects of the RM immune system remain ill characterized. In particular, B cell-mediated immunity in macaques has not been sufficiently characterized, and the B-cell receptor-encoding loci have not been thoroughly annotated. To address these gaps, we analyzed the circulating heavy- and light-chain repertoires in humans and RMs by next-generation sequencing. By comparing V gene segment usage, J-segment usage and CDR3 lengths between the two species, we identified several important similarities and differences. These differences were especially notable in the IgM^+^ B-cell repertoire. However, the class-switched, antigen-educated B-cell populations converged on a set of similar characteristics, implying similarities in how each species responds to antigen. Our study provides the first comprehensive overview of the circulating repertoires of the heavy- and light-chain sequences in RMs, and provides insight into how they may perform as a model system for B cell-mediated immunity in humans.

Rhesus macaques (RMs) are the most widely used non-human primate (NHP) model for biomedical research, and are commonly used for the study of microbial pathogenesis, host–pathogen interaction and drug or vaccine efficacy.^1, 2, 3, 4, 5, 6, 7, 8^ Research focused on human diseases such as Parkinson's, Alzheimer's, cancers and diabetes, as well as infectious diseases such as malaria, tuberculosis, Dengue virus, Ebola virus and HIV-1/AIDS utilizes RMs as a model organism because they are believed to closely approximate biological systems found in human beings.^1, 3, 5, 9, 10, 11, 12, 13^ In particular, RMs have become a widely used model to study disease progression and host–pathogen interactions in HIV-1, as they are naturally susceptible to infection by Simian Immunodeficiency Virus (SIV), the closely related progenitor of HIV-1.^14, 15^ The development of chimeric HIV-1/SIV-1 viruses (SHIVs), which encode an HIV-1 Env glycoprotein in an SIV genetic backbone, also have allowed the model to be used for comprehensive vaccination and viral challenge studies using the exact HIV-1 Env immunogens that would ultimately advance to human clinical trials.^16, 17, 18, 19, 20, 21, 22, 23, 24^ However, the use of RMs for preclinical vaccine studies remains somewhat problematic, as it is unclear how well the observed adaptive responses predict human immunity. In particular, B cell-mediated immunity is ill characterized in RMs. Thus, it is critical to understand how similar RM B cell-mediated immunity is to its human counterpart in order to assess the model's suitability for the preclinical evaluation of vaccines. Furthermore, it is important to identify where the two differ, so that the translation of preclinical experimental results in NHP to human clinical trials becomes more predictable.

The first completed RM draft genome was published in 2007 and enabled comprehensive comparisons between the macaque and human genomes, particularly those of the B-cell receptor (BCR)-encoding loci of each species.^25^ Macaques share 93% overall sequence identity and, like humans, maintain three BCR-encoding loci (on chromosomes 7, 13 and 10).^26^ However, significant changes have taken place since RMs and humans shared the last common ancestor—the IgH locus in humans and the IgK locus in RMs have expanded^25^—potentially altering the number of gene segments available for recombination. The use of the available RM genomic data is complicated by the incomplete annotation of the BCR loci and the presence of numerous sequence gaps (runs of N's).^25, 27^ In contrast, the human BCR-encoding loci (located on chromosomes 14, 2 and 22) are well annotated, allowing for straightforward lineage prediction for expressed immunoglobulin (Ig) sequences. In both organisms, the Ig heavy-chain (IgH) locus is organized as a tandem array of variable (V), diversity (D) and joining (J) gene segments. These segments recombine during B-cell maturation into a mature V–(D)–J rearrangement that expresses the mature heavy chain^28^ of the molecule. Similarly, the light-chain Ig kappa and lambda (IgK and IgL, respectively) loci are organized as arrays of V and J segments (there are no D segments in the light-chain loci) that recombine into a mature V–J rearrangement.^28^

The lack of coverage within the BCR-encoding genomic loci in RM has hampered efforts to understand the potential human context of vaccine-elicited B-cell responses, although there has been recent progress in identifying and evaluating potential BCR gene segments.^29, 30, 31^ One drawback of these studies is the lack of unity within the published segment nomenclature, which leads to redundancies and can be a source of confusion. A set of heavy- and light-chain gene segment sequences is available through the ‘Rhesus macaque Immunoglobulin gene database' (hosted by the King's College London School of Medicine), which is a collection of sequences from several sources and consists of 88 IGHV, 62 IGKV, 44 IGLV, 30 IGHD and 7 IGHJ segments. More recently, Sundling *et al.*^26, 30^ created an annotated list under a different nomenclature system, which consists of 63 IGHV, 62 IGKV, 50 IGLV, 30 IGHD, and 6 IGHJ, 5 IGKJ, 6 IGLJ gene segments. The international ImMunoGeneTics information system (IMGT) has begun to independently annotate the RM IgH locus, but there are currently 19 IGHV, 83 IGKV, 86 IGLV, 24 IGHD, 7 IGHJ, 4 IGKJ and 6 IGLJ gene segments available through this database.^32^ Finally, a provisional set containing 98 RM IGHV alleles with a new nomenclature was recently reported.^33^ Although it is uncertain that the available sets of reference sequences are exhaustive in coverage, they provide an indispensable foundation for annotating the circulating BCR repertoires in RM.

Until very recently, little was known about the makeup of the circulating B-cell repertoire in outbred RMs. Several recent studies utilized 454 pyrosequencing to define the circulating IGHV (heavy chain) gene family usage in IgG^+^ B-cell repertoires after vaccination or infection in a small number of animals.^31, 33^ These studies provided unique insight into the heavy-chain IgG^+^ BCR repertoires, but did not evaluate light-chain repertoires or the IgM heavy-chain repertoires. RM light-chain repertoires have only recently been described, but only in a single animal.^34^ The lack of available data describing IgM^+^ BCR repertoire is significant, as these B cells constitute the majority of the circulating BCR repertoire (~90% of circulating B cells in RMs), and are the cells that will initially respond to antigen after vaccination to give rise to the subsequent B-cell populations.^35^ These studies represented significant advances in our understanding of RM BCR repertoires, but the broad applicability of such findings to larger outbred RM populations and to human BCR repertoires remains unclear. This information can only be gained through an unbiased and systematic evaluation of the BCR-encoding sequences in a larger number of humans and RM, as we report here.

Understanding the genetic constitution of the circulating RM BCR repertoire is even more critical for vaccine studies aimed at eliciting antibody responses with specific genetic characteristics, such as studies aimed at eliciting broadly neutralizing antibodies (bNAbs) against HIV-1. These antibodies are well characterized in humans and are known to have common features and genetic requirements.^36, 37, 38, 39, 40, 41, 42, 43^ For example, a class of anti-CD4-binding site (CD4-BS) antibodies exemplified by the mAb VRC01 has a strong genetic restriction to the human IGHV1-2*02 allele and utilizes a CDRL3 of five amino acids (AAs).^41, 44, 45^ In addition, many bNAbs have long insertions in the third complementarity determining regions (CDR3's) in the heavy chains, which are thought to be assembled during VDJ rearrangement and are present in the naive circulating repertoires in humans.^46, 47, 48^ Both of these examples are characteristics that enable the BCR to bind to complex epitopes on the HIV Env glycoprotein and would need to be present in the circulating BCR repertoire in RMs in order to elicit such antibodies during preclinical vaccine studies.

We sought to compare the BCR repertoires in humans and RM in order to assess the utility and potential drawbacks of the RM model system for the study of B cell-mediated immunity. To accomplish this, we developed an NGS and bioinformatics platform based on Illumina sequencing technology. We sequenced both the IgM^+^ and IgG^+^ heavy-chain repertoires and both the IgK and IgL light-chain repertoires in five outbred RMs and five healthy human subjects using a 5' random amplification of cDNA ends (RACE) method. To minimize the overrepresentation by clonally-expanded sequences and to eliminate errors introduced by amplification and sequencing steps, we clustered sequences by V-segment family, J-segment family and CDR3 amino-acid sequences and used these data sets to represent the circulating repertoires. Side by side comparisons of the repertoires showed that the heavy-chain repertoires were similar in segment family usage between the IgG^+^ and IgM^+^ subsets within species, although some notable differences were observed in cross-species comparisons. Our comparisons of the IgK and IgL light-chain repertoires revealed that humans and RMs had moderately discordant gene family usage. Overall, our study provides the first comparative, comprehensive evaluation of BCR repertoires in RMs and humans that covers both the IgM (which are mostly naive B cells) and class-switched B-cell populations and includes all three BCR-encoding sequences. These analyses highlight important similarities and differences in the circulating BCR repertoires between humans and RMs that are relevant to their continued use as a preclinical model system for human B cell-mediated immunity research.

## Results

### A next-generation sequencing platform to assess BCR repertoires

The technologies to evaluate BCR repertoires by NGS have been advancing quickly over the last several years.^49^ And yet, a robust, easily deployed NGS platform to sequence full-length BCRs is not widely available. As such, we set out to develop a comprehensive pipeline for BCR repertoire analysis based on Illumina sequencing technology that could be easily adapted for use in a variety of species. This sequencing platform has several advantages that make it well suited for use in BCR sequencing, including long read lengths (up to 600 nucleotides) and relatively low error rates during the sequencing phase.^50, 51^

**Figure 1:**
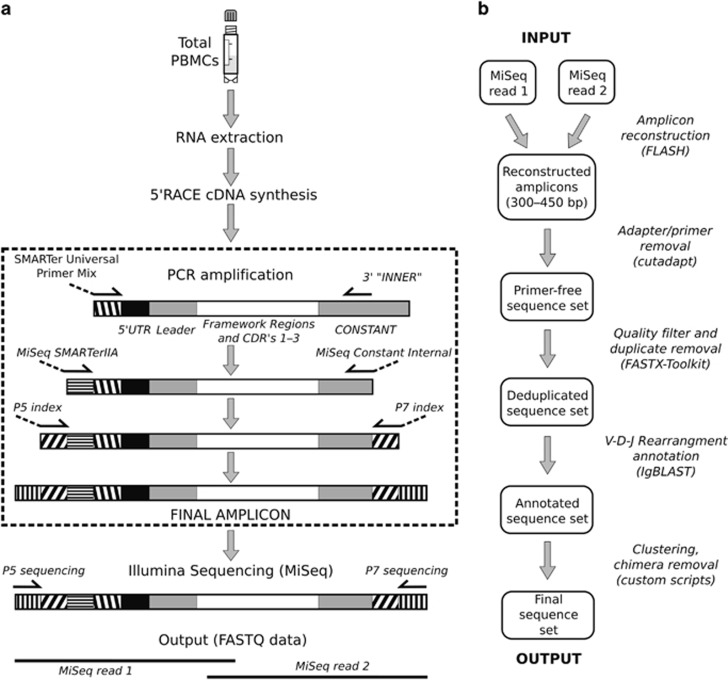
Sample preparation/analysis pipeline. Schematics depicting sample treatment and the PCR amplification steps required for library generation (**a**), and the computational pipeline used to filter and analyze the Illumina MiSeq output sets (**b**).

Our final platform utilized a modified 5' RACE method that enabled us to amplify and sequence the entire variable portions of the heavy and light chains in humans and RMs (Figure 1a). Of the various methods used for Ig library construction, 5' RACE is thought to produce the least biased libraries.^34, 52^ This is because it uses a single primer set (one universal and one gene-specific primer) for the amplification steps, rather than a multiplex or pooled primer method, in which a large number of primers may compete with one another during amplification. Therefore, it is likely that 5' RACE does not suffer from the amplification bias that accompanies the use of a large number of primer pairs.

For each of the three genetic loci in both humans and RM, we developed gene-specific primers for the initial cDNA first-strand synthesis, rather than using a polydeoxythymidine primer (oligo-dT). For PCR amplification steps, we developed custom reverse primers that were nested inside the synthesis primer (Supplementary Table S1). For the forward PCR primer, we utilized a universal primer in all reactions. The final step in library production was the addition of Illumina compatible linker sequences by a PCR refurbishing step that allowed capture of PCR products on the sequencing chip. Our reverse Illumina adapter primer was further nested inside the amplification primer, creating a protocol with two semi-nested amplification steps (Figure 1a). Within the IgH sequences, the reverse primers provided specificity between the IgM^+^ and IgG^+^ subsets. For IgM and IgG, the final PCR amplicons were ~550–650 bp in length, whereas the IgK and IgL amplicons were 500–600 bp long. Each amplicon covered the entire V–(D)–J rearrangement (all of framework regions (1–4) and CDRs (1–3)) and a portion of the 5′ UTR. The sequencing primers used by MiSeq generated forward and reverse reads (‘MiSeq read 1' and ‘MiSeq read 2', respectively, in Figure 1a) that overlapped in the middle of the amplicon, and allowed subsequent amplicon sequence elucidation (see below).

The raw FASTQ sequence data harvested from a sequencing run underwent a number of processing steps before comparative repertoire analysis. In the absence of a readily available informatics toolkit, we assembled an informatics pipeline that used currently available academic programs to facilitate the processing, finishing and quality control of each data set (detailed in Materials and Methods, and in Figure 1b). Raw sequences from forward and reverse reads were first matched to creating a single extended (forward-orientation) sequence, followed by the elimination of any reads in which the primer sequences could not be identified. Additional quality control steps discarded low-quality sequences, and coalesced duplicate sequences. Finally, the sequence sets were annotated with V, D and J segments usage and grouped into sequence clusters (based on CDR3 amino-acid sequence, V-segment family and J-segment family annotations, see below) in order to (1) identify and remove suspected chimeric sequences, (2) eliminate potential overrepresentation by dominant B-cell clones and (3) eliminate errors introduced by amplification and sequencing steps. The population diversity found within these clustered data sets is described in Supplementary Figure S1.

A comprehensive Ig gene segment reference set for RM was not available, necessitating the construction of a sequence database containing all of the previously-identified V, D and J segments. The published *Macaca mulatta* genome, as well as other sequencing efforts, has resulted in several publically available compilations of Ig heavy-chain sequences. The majority of the current genome sequence was obtained from one or two animals;^25^ with the remaining identified sequences originating from a collection of different genomic scaffolds,^53^ locus sequencing efforts^33^ as well as sequences documented by the Rhesus macaque Immunoglobulin database (accession numbers DQ437773–855, AY161053–81, AF173903–32, AF417167–96, U57580–7, AY057983, AY963709–73 and AM055970–6022). In absence of fully annotated genomic loci, it is currently difficult to reliably estimate the total number of germline gene segments. However, in order to utilize all available RM Ig information, we compiled all of the available non-redundant germline heavy- and light-chain gene segments for our analyses.^30, 31, 33^ A list of germline gene segments identified according to their original designation, accession number, alternative nomenclature and closest human germline gene segment homolog is shown in Supplementary Table S2.

### IGHV gene segment family usage in the circulating IgM and IgG BCR repertoires

**Figure 2:**
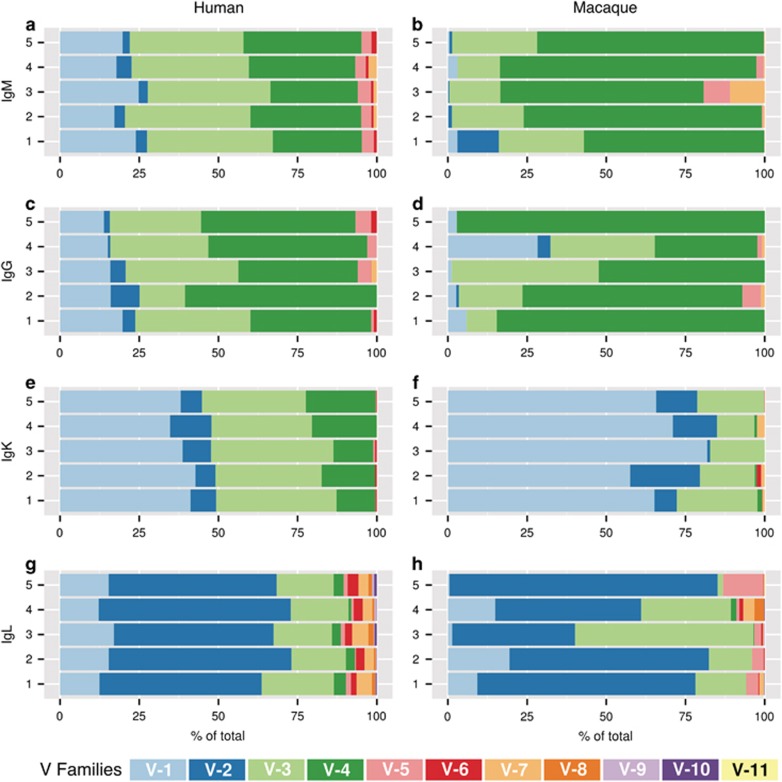
Distribution of V-family genes in the expressed IgM^+^ (**a**,**b**), IgG^+^ (**c**,**d**), IgK^+^ (**e**,**f**), and IgL^+^ (**g**,**h**) BCR repertoires of healthy human subjects (**a**,**c**,**e**,**g**) and untreated macaques (**b**,**d**,**f**,**h**). Productively rearranged sequences amplified from the expressed BCR repertoires for each human subject (*n*=5) or animal (*n*=5) were grouped into sequence clusters and are represented by horizontal bars. V-family assignments are color-coded (color assignments are described below the graphs) and scaled according to the proportion present in each data set.

We evaluated the assigned IGHV segment family usage within the IgM sequence libraries and compared the frequencies of segment family usage between humans and RMs (Figures 2a and b). To rule out an overestimation of IGHV gene segment usage within the different families due to clonal expansion, we focused our analysis on sequence clusters, rather than including all non-redundant sequences. Our clustering strategy grouped sequences with closely related V–(D)–J segment rearrangements; so that sequences with identical CDR3 amino-acid sequence and using V and J segments belonging to the same families were coalesced to form a single sequence cluster. Only clusters containing five or more sequences were included in the repertoire data sets. In this way, our system is able to correct for errors and reduce the potential bias in gene family usage that could be caused by the presence of a large number of closely related sequences, resulting from a few nucleotide differences due to PCR or sequencing error, or from somatic mutation in highly stimulated B-cell lineages. However, we also analyzed the non-redundant, non-clustered sequence sets to evaluate for sequence bias before clustering (Supplementary Figure S2). In all of the non-redundant sequence sets, the gene family usage approximated that of the clustered sequence sets, indicating that our results were likely an accurate representation of the actual circulating BCR repertoires in both species and that the gene family distributions were not skewed due to over-abundant, closely related sequences that could have arisen from clonal expansion or sequencing errors.

In humans, the IGHV3 and IGHV4 gene families were the predominantly expressed segment families, present at ~38 and 32%, respectively (Figure 2a, Supplementary Figure S4A). The IGHV1 family was the next most abundant (~21%), with the remainder of the gene families combining to make up <9% of the repertoires (Figure 2a). In contrast, the RM IgM compartment was dominated by IGHV4 gene family, making up ~70% of the expressed IgM sequences (Figure 2b). IGHV3 was the next most abundant, averaging ~21%, whereas the remaining gene families IGHV1, 2, 5 and 7 combined to make up only ~9% of the expressed repertoires in macaques. We did not detect any IGHV6-family segment usage in macaques using 5' RACE amplification. However, IGHV6 transcripts were detectable in a standard RT-PCR containing IGHV6-specific primers (data not shown). Thus, it is likely that IGHV6-containing transcripts in macaques are present, but at an extremely low abundance. Comparatively, human repertoires contained significantly more IGHV1 (*P*<0.0001) and IGHV3 (*P*<0.0001) than RM repertoires, whereas RMs contained more IGHV4 (*P*<0.0001) (Supplementary Figure S4). Thus, the circulating IgM^+^ repertoires in humans and RMs differed significantly in the three most prevalent gene families.

We also evaluated the IGHV segment family usage in circulating IgG^+^ B cells in both humans and RMs. The IgG^+^ repertoire represents a population of B cells that have experienced antigen stimulation, have undergone additional development, class-switching and proliferation, and are poised to rapidly respond to secondary antigenic stimulation during repeat infection.^54^ In comparing the IgG^+^ compartments, humans and RMs expressed very similar IGHV gene family repertoires, with the exception of IGHV4, which was more abundant in RM IgG repertoires (*P*=0.013) (Supplementary Figure S4). Within each species, we also compared the IGHV gene family distribution between the IgG and IgM repertoires. In humans, the IgG repertoires contained more IGHV4 (*P*<0.0001) and less IGHV3 (*P*=0.0058) than the IgM repertoires, indicating that IGHV4 may be more prominently stimulated into B-cell memory (Supplementary Figure S4). In macaques, the IgG and IgM repertoires expressed very similar distributions of IGHV families, with no statistically significant differences noted between the two B-cell compartments (Supplementary Figure S4).

### Divergent V-segment family usage in the IgK, but not IgL, repertoires between RMs and humans

A mature BCR consists of one heavy chain encoded by the IgH locus and one light chain encoded by either the IgK or IgL locus. In humans, the IgK repertoires predominantly consisted of the IGKV1 and IGKV3 gene segment families, which combined accounted for nearly 75% of the repertoires in most of the subjects (Figure 2e). The IGKV4 and IGKV2 gene families accounted for ~16 and 8% of the human repertoires, respectively. The remaining gene families accounted for the remaining 1%, although we did not detect expression of IGKV7 genes in humans. In contrast, all IGKV gene families were detected in RMs (Figure 2f). The IgK repertoires in RM were predominantly IGKV1 (68%). The IGKV3 and IGKV2 families accounted for 18 and 11% of the repertoires, respectively, with the remaining gene families accounting for the remaining 3%. Comparatively, the human IgK repertoires expressed more IGKV3 (*P*>0.0001) and IGKV4 (*P*>0.0001) than RMs, whereas the RM repertoires contained significantly more IGKV1 (*P*>0.0001) (Supplementary Figure S4).

Interestingly, the IgL repertoires were remarkably similar between the two species. Both repertoires predominantly expressed the IGLV2, accounting for ~55% in humans and 60% in RM (Figures 2g and h). IGLV3 was the next most prevalent gene family in both species (19% in humans and 23% in RM), followed by IGLV1 (14% in humans and 9% in RMs). We did not detect IGLV11 gene family expression in humans, and detected it in only two out of five RMs at very low levels (<0.1%). The remaining seven IGLV gene families were present at <1–3% in the repertoires. Comparatively, we noted no statistically significant differences in the IgL gene family distributions between humans and RM (Supplementary Figure S4).

### Humans and RMs express similar J-gene segment family distributions, with the exception of IgL

**Figure 3:**
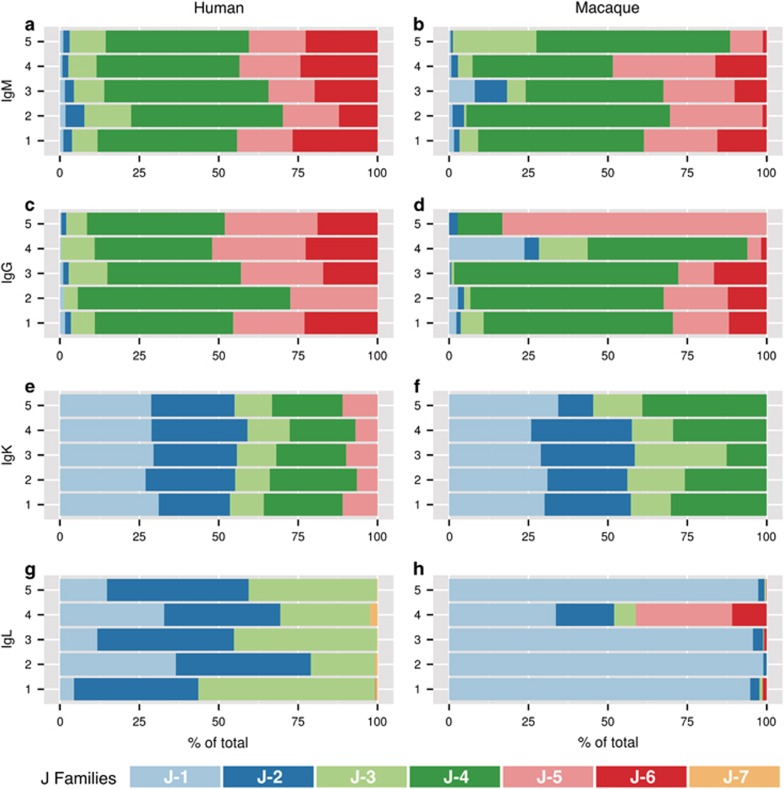
Distribution of J-family genes in the expressed IgM^+^ (**a**,**b**), IgG^+^ (**c**,**d**), IgK^+^ (**e**,**f**), and IgL^+^ (**g**,**h**) BCR repertoires of healthy human subjects (**a**,**c**,**e**,**g**) and untreated macaques (**b**,**d**,**f**,**h**). Productively rearranged sequences amplified from the expressed BCR repertoires for each human subject (*n*=5) or animal (*n*=5) were grouped into sequence clusters and are represented by horizontal bars. J-family assignments are color-coded (color assignments are described below the graphs) and scaled according to the proportion present in each data set.

We also compared J-family usage for all three BCR-encoding sequences between humans and RMs. The J-gene segment is assembled into the V–(D)–J (or V–J) rearrangement during B-cell maturation and comprises part of the CDR3 at the D/J or V/J boundary. We found a high degree of similarity in the IGHJ gene segment family distributions between humans and RMs in three of the four repertoires we analyzed (Figure 3; compare with Supplementary Figure S3 for unclustered data). Heavy-chain sequences of both the IgG and IgM repertoires were dominated by IGHJ4 in humans and RMs (Figures 3a–d). IGHJ5 and IGHJ6 were the next two most commonly expressed segment families, although IGHJ6 was more heavily expressed in humans than IGHJ5 (Figures 3a and b), whereas RMs expressed more IGHJ5 than IGHJ6 (Figures 3b and d). The remaining IGHJ segment families together constituted an average of <10% of the overall repertoires. Comparatively, the only difference observed was that humans expressed more IGHJ6 than RM (*P*=0.0086) (Supplementary Figure S5). Thus, although significant differences in IGHV gene usage were observed between humans and RMs, these differences were not reflected in the incorporation of J-gene segments.

Similarly, the IGKJ gene segment family usage was remarkably concordant between humans and RMs (Figures 3e and f), despite the major differences we observed in IGKV segment usage (Figures 2e and f). IGKJ families 1, 2 and 4 were the most commonly expressed gene segment families in both species. Overall, the distribution of IGKJ gene usage was statistically similar between species with one exception: humans expressed more IGKJ5 than RMs (*P*=0.0276) (Supplementary Figure S5). In contrast, IGLJ segment family expression between RMs and humans was discordant (Figures 3g and h), despite the observation that the IGLV gene usage between species was statistically similar (Figures 2g and h). The IGLJ2 family was the most abundantly expressed segment family in humans, followed by the IGLJ1 and IGLJ3 families (Figure 3g). In RM, the IGLJ repertoires were predominantly IGLJ1 (Figure 3h). Comparatively, RMs expressed more IGLJ1 than humans (*P*<0.0001), whereas humans expressed more IGLJ2 (*P*<0.0001) and IGLJ3 (*P*<0.0001) than RMs (Supplementary Figure S5).

### Analysis of CDR3 sequence lengths

**Figure 4:**
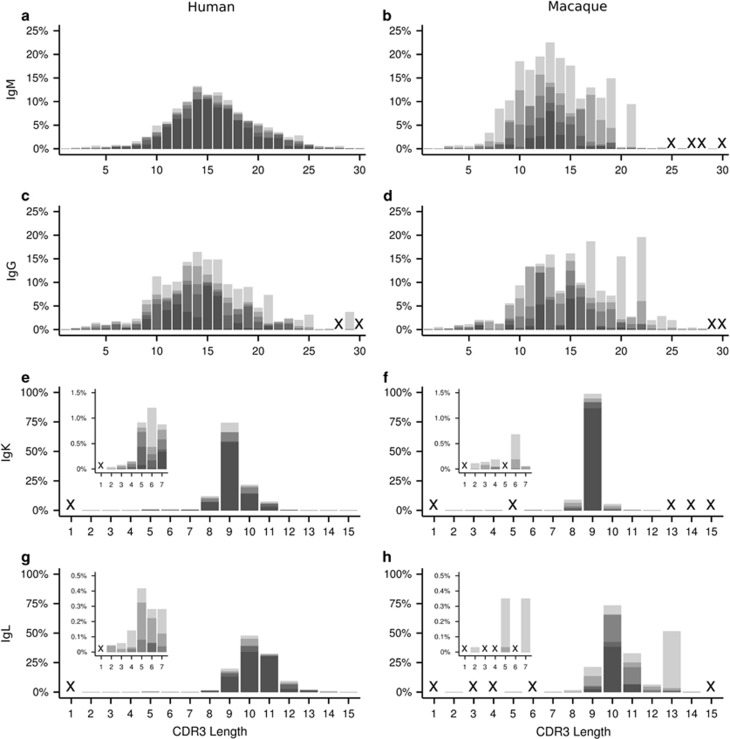
Distribution of CDR3 lengths in the expressed IgM^+^ (**a**,**b**), IgG^+^ (**c**,**d**), IgK^+^ (**e**,**f**), and IgL^+^ (**g**,**h**) BCR repertoires of healthy human subjects (**a**,**c**,**e**,**g**) and untreated macaques (**b**,**d**,**f**,**h**). Lengths of CDR3 amino-acid sequences were compiled for each of the human (*n*=5) and macaque (*n*=5) data sets and plotted as stacked transparent gray bars. Darker regions of the plots indicate occurrence in multiple data sets. Very low abundance outlier sequences (CDR3 lengths >30 AAs for VH, and >15 AAs for VL) were excluded from the plots. Short (<8 AAs) CDRL3 sequences present at lower frequencies are shown as insets (**e**–**h**). An ‘x' denotes that no sequences were found containing CDR3s of that length.

For heavy-chain repertoires, the average length of CDRH3s in humans and RMs in both the IgM and IgG sequence sets was similar, averaging 13–15 AAs in length (Figures 4a–d). However, humans and RMs did not generate long CDRH3s (⩾20 AAs in length) with similar frequencies. Long CDRH3s in the IgM sequences were more frequently detected in humans than in RMs (*P*=0.0005, Supplementary Figure S6), with an average of 15.5% (±1.32%) of human IgM repertoires consisting of sequences with long CDRH3s, compared with 2.49% (±1.92%) in RMs. However, this is likely an overestimation for RMs, as one animal out of five had an unusually large number of sequences with long CDRH3s (~10% in the IgM) (Figure 4b, Supplementary Figure S6). Excluding this one animal, RMs averaged only 0.52% (±0.16%) long CDRH3. Interestingly, within in the IgG sequence sets the prevalence of long CDRH3 was not statistically different between humans and RMs (*P*=0.58), with the human sequence sets containing an average of 14.79% (±2.18%) and RMs an average of 16.72% (±2.5%). Thus, although CDRH3 lengths of ⩾20 AA were rare in the IgM^+^ B-cell compartment in RMs, they appeared to populate the class-switched B-cell compartment at a similar rate to that of humans.

In the light-chain repertoires, humans and RMs expressed BCRs with the same average CDRL3 lengths (Figures 4e–h, Supplementary Figure S6). In both species, the average CDRL3 length in the IgK populations was 9 AAs, and 10 AAs in the IgL repertoires. Long CDRL3s (⩾13 AAs in length) were rare in both humans and RMs in the IgK sequence sets, making up <1% of the repertoires (Figures 4e and f, Supplementary Figure S6). In the IgL sequences, long CDRL3 was slightly more common, averaging ~2% of the repertoires (Figures 4g and h, Supplementary Figure S6). Short CDRL3s (⩽7 AAs in length) were also rare in the IgK and IgL repertoires, making up <0.3–0.6% of sequence populations. Interestingly, an exception was the set of human IgK repertoires, which averaged 1.74% (±0.36%) short CDRL3 (Figure 4e, inset, Supplementary Figure S6). Comparatively, humans expressed significantly more short CDRL3s than RMs (*P*=0.0062, Supplementary Figure S6).

### Key sequence features within the BCR repertoires relevant to HIV-1 vaccine development

As noted above, some classes of anti-HIV-1 bNAbs have common defining characteristics that are essential to their broadly neutralizing activities, namely CDR3 length and genetic restriction. The collection of comparative NGS data sets on the BCR-encoding loci in humans and macaques provides an opportunity to evaluate the prevalence of these characteristics in the circulating BCR repertoires. As described above, we evaluated the BCR repertoires for the presence of long CDRH3s, which are a common characteristic of many bNAbs. We found that RMs express long CDRH3s in the IgM BCR populations with low frequency, whereas they are much more frequent in humans (around 15% of a repertoire, Supplementary Figure S6). However, in the IgG repertoires, RMs expressed long CDRH3s at a rate similar to humans, around 15% of the total IgG repertoires. This suggests that the rarity of IgM^+^ B cells with long CDRH3s does not preclude their significant contribution to the memory compartment in RM. Thus, RMs are well suited to produce secreted antibodies with long CDRH3s. Indeed, monoclonal antibodies with long CDRH3s have been isolated from RMs previously.^33, 55^

We also evaluated the BCR repertoires for two key features reported to be important for the development of the VRC01 class of anti-CD4-BS bNAbs: IGHV allelic restriction and an IgK CDRL3 of five AAs in length.^41, 44, 45^ Five-AA CDRL3s in the IgK repertoires were detected in all of the humans that we analyzed, making up 0.6% (95% CI [0.17,1.02]) of the total IgK repertoire (Figure 4e, inset). In contrast, we did not detect 5-AA CDRL3s in the IgK repertoires in any of the macaques (Figure 4f, inset). Thus, IgK 5-AA CDRL3s are likely so extraordinarily rare that they are not readily captured in routine unbiased analysis.

Additionally, VRC01-class bNAbs are known to be restricted to a specific human IgH allele, IGHV1-2*02.^41, 44, 45^ This is thought to be due to the presence of three key AAs encoded in the germline sequence that interact with the HIV-1 Envelope protein, amino-acid positions W50, N58 and R71.^44^ The IGHV1-2*02 allele was found to be expressed in four of five humans in this study at an average of 4.43% (95% CI [3.26,5.61]) of the total BCR heavy-chain repertoires (Supplementary Figure S7). This allele was also detected in the remaining human subject, but at extremely low frequency (0.0041%). IGHV1-Kl (Supplementary Table S2), which is also known as VH1.23,^56^ is the closest homologous sequence in RMs to the human IGHV1-2*02 at 92% amino-acid sequence identity. Despite its close sequence relatedness, IGHV1-Kl encodes for only two of the three key AAs needed for VRC01 binding, and lacks a tryptophan at position 50. IGHV1-Kl was detected in 4/5 animals at low levels, with an average frequency of 0.62% (95% CI [−0.66,1.91]) (Supplementary Figure S7). These data imply that humans are well equipped within their IgM^+^ B-cell compartments to develop VRC01-like antibodies with these characteristics. However, the RM homolog of IGHV1-2*02, which is missing a key amino-acid position, was far less abundant, and RM expression of IgK chains with five-AA CDRL3s was not detectable. Thus, RMs likely are not competent to develop antibodies with the characteristics typical of VRC01-class antibodies.

## Discussion

Given the importance of RMs for biomedical research, we sought to characterize the expressed BCR heavy- and light-chain repertoires in RMs and systematically compare them to human expressed BCR repertoires. Our goal was to provide foundational data that can be used by researchers to make rational assessments about the utility of RMs as a model system to study B cell-mediated immune response in humans. We developed an Illumina sequencing technology-based NGS B-cell analysis pipeline based on the bulk amplification of the expressed V–(D)–J (or V–J) rearrangements of the three BCR-encoding loci (IgH, IgK and IgL) from the IgM and IgG heavy-chain repertoires, and examined samples from five humans and five RMs in parallel. Concurrent sequencing of the four BCR chain repertoires separately in each subject enabled us to conduct a quantitative, population-based comparison. Our analyses included both V- and J-segment usage in the heavy- and light-chain repertoires, as well as CDR3 length comparisons. Our study provides the first comprehensive, comparative evaluation of the B cell heavy- and light-chain repertoires in humans and in outbred RMs, and provides insights about the translational potential of experimental results to human B cell-mediated immunity.

Characterization of BCR repertoires with unprecedented depth is now possible due to advances in NGS. Here, we report one of the most broad and detailed analyses of BCR repertoires to date in either humans or RMs, which allows us to compare repertoires among the individuals and between the species. In humans, the frequency of V- and J-gene segment usage among human subjects in the IgM, IgG, IgK and IgL repertoires was in good agreement, as were the distributions of CDR3 lengths. Similarly, with a few exceptions, the repertoires and CDR3 lengths were concordant among the individual RMs, especially in the IgM, IgK and IgL repertoires. The relative similarity of the BCR repertoires in individual subjects between and within species is remarkable, considering that our data represent snapshots of continuously changing populations of B cells that arose in unique immunological circumstances in each individual. In addition, the repertoires that we report here are in good agreement with those reported in previous studies for both humans and RMs.^34, 40, 52, 57, 58, 59, 60, 61^ Taken together, these findings provide confidence that the BCR repertoires we report here are representative of actual average circulating repertoires in both species, although the collection of more repertoire data will be needed to confirm this.

Comparative analyses of the IgM, IgG, IgK and IgL sequences in RMs and humans revealed some significant differences in the overall makeup of the circulating B-cell repertoires. Namely, gene segment family usage and the frequencies of CDR3s with lengths at the extremes of the size distribution differ moderately between RMs and humans in the IgM^+^ B-cell compartment. Nevertheless, it may be that these differences are too subtle to have significant functional consequences for the utility of RM as a model system. Importantly, we found that (1) RMs express diverse BCR repertoires that contained sequences utilizing nearly all of the V- and J-gene families in both the heavy and light chains, (2) they possessed a significant range of CDR3 lengths and (3) these parameters were generally comparable to those we found in humans. Given the observed recombinatorial complexity and diversity of the B-cell repertoires in RMs and the close genetic relationship between the two species, it is likely that RM B-cell populations are poised to respond to antigen in ways similar to those of humans.

Indeed, the differences in V- and J-family distributions in the IgM^+^ repertoires between humans and RMs largely resolved into concordant gene family usage in the class-switched (IgG) compartment (Supplementary Figure S4B). This convergence was not due to a remodeling in the gene family distribution in RMs, but rather reflected changes in gene family frequencies between the IgM and IgG repertoires in humans (Supplementary Figures S4E and F). Additionally, the low abundance of long CDRH3s in RM IgM^+^ B cells did not preclude their relative expansion in the IgG^+^ population, and they were present to a similar degree to human IgG repertoires (Supplementary Figure S6A). Thus, both the human and RM IgM populations underwent some degree of repertoire remodeling after antigen education, but converged on a set of similar characteristics that defined the IgG^+^ B-cell populations. This level of convergence is remarkable, considering that the changes between the IgM and IgG cell populations in each species are driven by different natural histories of antigen exposure. Taken together, these observations imply that RMs possess the capacity to recapitulate a class-switched memory B-cell compartment similar to that of humans and are likely suitable model organisms for the study of B cell-mediated immunity.

Despite the general similarity, there are likely rare cases in which the RM model will not provide an adequate facsimile of human immunity, as described above for the VRC01-class of anti-HIV-1 antibodies. As such cases arise, in which specific sequence characteristics are necessary to gain the desired immune response, the competency of RMs to recapitulate these responses should not be simply assumed and should be evaluated directly. If these characteristics are genetic in nature, then continued sequencing efforts targeting the RM BCR-encoding loci will be required to evaluate the suitability of RM as a model. Nevertheless, such shortcomings do not imply that RMs are an inadequate model system for the study of HIV-1 bNAbs targeting in or around the CD4-BS in general, as bNAbs that do not use IGHV1-2*02 or do not require 5-AA CDRL3s have been described in HIV-1-positive humans.^44, 45, 62, 63^ Further, the comparable abundance of BCRs with long CDRH3s in the RM IgG populations indicates that RMs can recapitulate that particular aspect of many anti-HIV-1 bNAbs. Indeed, bNAbs with similar specificities to those of humans have been isolated from RMs in previous studies, supporting the continued use of RMs as a model for the study of HIV-1 bNAbs.^31, 34, 56, 64, 65^

However, there are caveats associated with B-cell repertoire analysis by NGS. Although we have evaluated both the heavy- and light-chain repertoires, we did not have the ability to pair up natively-matched heavy- and light-chain pairs. Thus, we cannot say what fraction of the IgK and IgL repertoires comes from the memory B-cell compartment and we cannot evaluate the important dimension of the frequency of specific heavy- and light-chain pairings. Methods to do such analyses are now becoming available, but they are not widely used and their use for comprehensive, high-throughput analyses is not yet practical. Further, the humans included in this study were a mix of male and females, but they are all of North American origin. Recent evidence suggests far more geographic IGHV allelic variation than was previously thought.^66^ Additionally, the makeup of B-cell repertoires can be influenced by sex and age, and although the animals used in this study were approximately the same age and life stage of the humans, the differences in actual age may confound our analysis. Reporting the repertoires as gene family (as we have done here) likely mitigates these variations, but inclusion of geographically disparate samples and larger age ranges in subsequent studies will be a necessary future step. Finally, lack of allelic coverage in RMs continues to be an issue in NGS analyses, although efforts are under way to further characterize the IgH locus.

Overall, our studies provide a comprehensive overview of the circulating IgM, IgG, IgK and IgL repertoires in outbred RMs, allowing for the first time an extensive global comparison with the circulating B-cell repertoires in humans. Our study revealed many striking similarities, indicating that RMs are a reasonable model system for human B cell-mediated immunity. However, our study also revealed several differences that may be relevant to the performance of RMs in specific cases. Further characterization of the BCR-encoding loci and functional analyses of B-cell repertoires will serve to clarify exact relationships between the humans and RMs, and allow for more extensive analyses of the translatability of experimental results to humans.

## Methods

### Sample acquisition

Whole blood samples were obtained by venipuncture from five human subjects under CIDR human subjects protocol HS029, which was reviewed and approved by the Western Institutional Review Board. In addition, samples were obtained by venipuncture from five RMs of Indian origin under IACUC-approved protocols. The human subjects ranged in age from 22 to 32 years and were a mix of males and females. The macaques were all sexually mature males aged 4–6 years. Adjusted for relative lifespan, the humans and macaques in this study were roughly of the same life stage. Peripheral blood mononuclear cells (PBMCs) were isolated from whole blood by Ficoll density gradient separation. After two washes in RPMI containing 10% fetal bovine serum, PBMCs were slowly frozen in fetal bovine serum (Sigma, St Louis, MO, USA) supplemented with 10% DMSO in Mr. Frosty (Thermo Fisher Scientific, Waltham, MA, USA) cryo-containers. The RMs were housed at the Washington National Primate Research Center in Seattle, WA, USA. The care and all procedures were carried out under approved protocols monitored by the University of Washington Institutional Animal Care and Use Committee (IACUC).

### Cell preparation

Macaque PBMCs were briefly thawed at 37 °C, diluted 10-fold in RPMI and centrifuged for 10 min at 400 *g*. Cells were washed once in phosphate-buffered saline, centrifuged again, resuspended in the FACS buffer (phosphate-buffered saline w/ 2% fetal bovine serum) and enumerated using a Countess II FL cell counter (Thermo Fisher Scientific). Cells were then resuspended in Buffer RLT (Qiagen, Germantown, MD, USA) and stored at −80 °C until RNA was extracted.

### Library preparation for Illumina MiSeq

B-cell libraries for Illumina MiSeq sequencing were prepared, as follows. Total RNA was extracted from 5 to 10 million PBMCs using the AllPrep DNA/RNA Mini Kit (Qiagen). In an effort to generate unbiased B-cell libraries, cDNA synthesis was subsequently performed using the Takara Clontech SMARTer RACE cDNA Amplification Kit using primers with specificity to IgG, IgM, IgK and IgL. The subsequent RACE-ready cDNA was diluted in Tricine-EDTA according to the manufacturer's recommended protocol. First-round Ig-encoding sequence amplification was performed using AccuPrime *Pfx* Supermix (Invitrogen, Waltham, MA, USA), containing gene-specific primers (120 nm) and 1 × concentration of Takara/Clontech 10 × Universal primer mix. Amplicons were purified using FlashGels (Lonza, Allendale, NJ, USA) and used as templates for second-round PCR amplification. A second-round PCR amplification (10 cycles) was performed in order to add MiSeq adapter sequences to both ends of the amplicon. After re-purification, a final 5-cycle amplification was performed by adding P5 and P7 index sequences for Illumina sequencing. Purified, indexed libraries were quantitated using the KAPA library quantification kit (Kapa Biosystems, Wilmington, MA, USA) performed on an Applied Biosystems 7500 Fast real-time PCR machine (Applied Biosystems, Foster City, CA, USA).

### Illumina MiSeq operation

Libraries were denatured and loaded onto Illumina 600-cycle V3 cartridges, according to the manufacturer's suggested workflow. Briefly, libraries were combined at equimolar concentrations, denatured and neutralized according to the manufacturer's protocol for 5 min at room temperature with freshly prepared 0.2 N NaOH. After incubation, the reaction was diluted with ice-cold, HT1 Hybridization Buffer (Illumina, San Diego, CA, USA). Illumina PhiX Control (12.5 pm) was used as an internal quality control according to the manufacturer's protocol at a concentration of 5% of the final, combined library volume.

### Sequence analysis of human and RM IgH, IgL and IgK libraries

Raw data obtained from the forward and reverse MiSeq reads were used to reconstruct the amplicon using FLASH.^67^ The resulting sequence sets were filtered to select only sequences that contained the amplification primers (a procedure during which the primer sequences themselves were removed) using cutadapt;^68^ sequences containing low-confidence base calls (N's) were then removed from the set using FASTX-toolkit (http://hannonlab.cshl.edu/fastx_toolkit). Annotation of the resulting sequence sets was carried out using IgBLAST^69^ against a customized database of macaque gene segment sequences listed in Supplementary Table S2 or against a similar human data set.

The IgBLAST annotation was used for identification of the closest-matching V and J segments for each sequence. D-segment annotations were excluded from this analysis due to the low confidence of alignment of such short sequences (frequently, this problem manifested as multiple D-segment matches with identical scores). CDR3 sequences were identified in the heavy-chain amino-acid sequences using the following strategy: sequences were scanned for an anchor sequence Y(Y/H/F)C (indicating the end of Framework 3) followed by an anchor sequence WGxG (with ‘x' denoting any residue), and, failing that, a W alone; the sequence between the anchor sequences was harvested as CDRH3. Similarly, light-chain sequences were scanned for Y(Y/H/F)C and FG as anchor sequences, and the intervening sequence was harvested as CDRL3. Annotated sequences that were determined to be productively rearranged (that is, lacking stop codons and in-frame with the J-segment sequence) and containing the consensus motifs of Framework 4 (at least 30 000 sequences per data set) were grouped together based on identical CDR3 amino-acid sequences and defined as sequence clusters. This procedure was further used to identify and filter away artifactual chimeric sequences, which differed in V-family and/or J-family assignment despite sharing the CDR3 sequence with their putative clusters. Furthermore, clustering was used to counteract potential dominance of expanded B-cell clones, and to eliminate sequence errors that arose during amplification and sequencing. We used the R-package *alakazam*^70^ in order to characterize clustered populations by calculating the Hill diversity indices, as well as Shannon's entropy and evenness metrics (Supplementary Figure S1).

### Statistical analyses

All statistical analyses were performed in GraphPad Prism 6.07 statistical software package (GraphPad Software Inc., La Jolla, CA, USA). The comparison of IgV and IgJ gene family frequency between humans and RMs was carried out using two-way ANOVA. Multiple comparisons were carried out using Sidak's multiple comparisons test (Alpha=0.05) with Sidak's correction for multiple comparisons. Reported *P-*values are corrected for multiple comparisons. Characteristics of the CDRH3 and CDRL3 populations were compared by unpaired *t* test, assuming equal standard deviations (parametric). *P*-values of <0.05 were considered as significant. In those cases where averages are reported from the *t* test groups or two-way ANOVA, we also report the standard error of the mean (SEM), as denoted by the addition of (±*X*%). In the figures describing statistical analyses, the error bars are the standard deviation. Simple averages in which the means between two populations were not compared statistically are reported as the average percent and the 95% confidence interval.
