# Supplementary material for: Repertoire comparison of the B-cell receptor-encoding loci in humans and rhesus macaques by next-generation sequencing
Source: Clin Transl Immunology. 2016 Jul 22;5(7):e93–. doi: 10.1038/cti.2016.42 (PMC4973324; doi:10.1038/cti.2016.42)
Supplement: Supplementary Information [file cti201642x1.pdf]

Supplementary materials:

Fig. S1. Population structure of the sequence sets following clustering by V-family, J-family and CDR3 amino acid sequence.

Fig. S2. Distribution of V-family genes in the non-redundant (non-clustered) expressed BCR repertoires of healthy human subjects and untreated macaques.

Fig. S3. Distribution of J-family genes in the non-redundant (non-clustered) expressed BCR repertoires of healthy human subjects and untreated macaques.

Fig. S4. Statistical analysis of the IgV gene family frequencies in the IgM, IgG, IgK, and IgL repertoires of humans and rhesus macaques.

Fig. S5. Statistical analyses of the J gene family frequencies in the IgM, IgG, IgK, and IgL repertoires of humans and rhesus macaques.

Fig. S6. Statistical analyses of the CDRH3 and CDRL3 lengths in IgM, IgG, IgK, and IgL repertoires of humans and rhesus macaques.

Fig. S7. Frequency of IGHV1-2*02 gene in human BCR repertoires and its rhesus homolog IGHV1-KI in rhesus macaque BCR repertoires.

Table S1. Primer sets used in the construction of NGS libraries for humans and rhesus macaques

Table S2. Unified Macaque Immunoglobulin Segment Library (electronic format).

Supplementary Figure 1. Population structure of the sequence sets following clustering by V-family, J-family and CDR3 amino acid sequence.

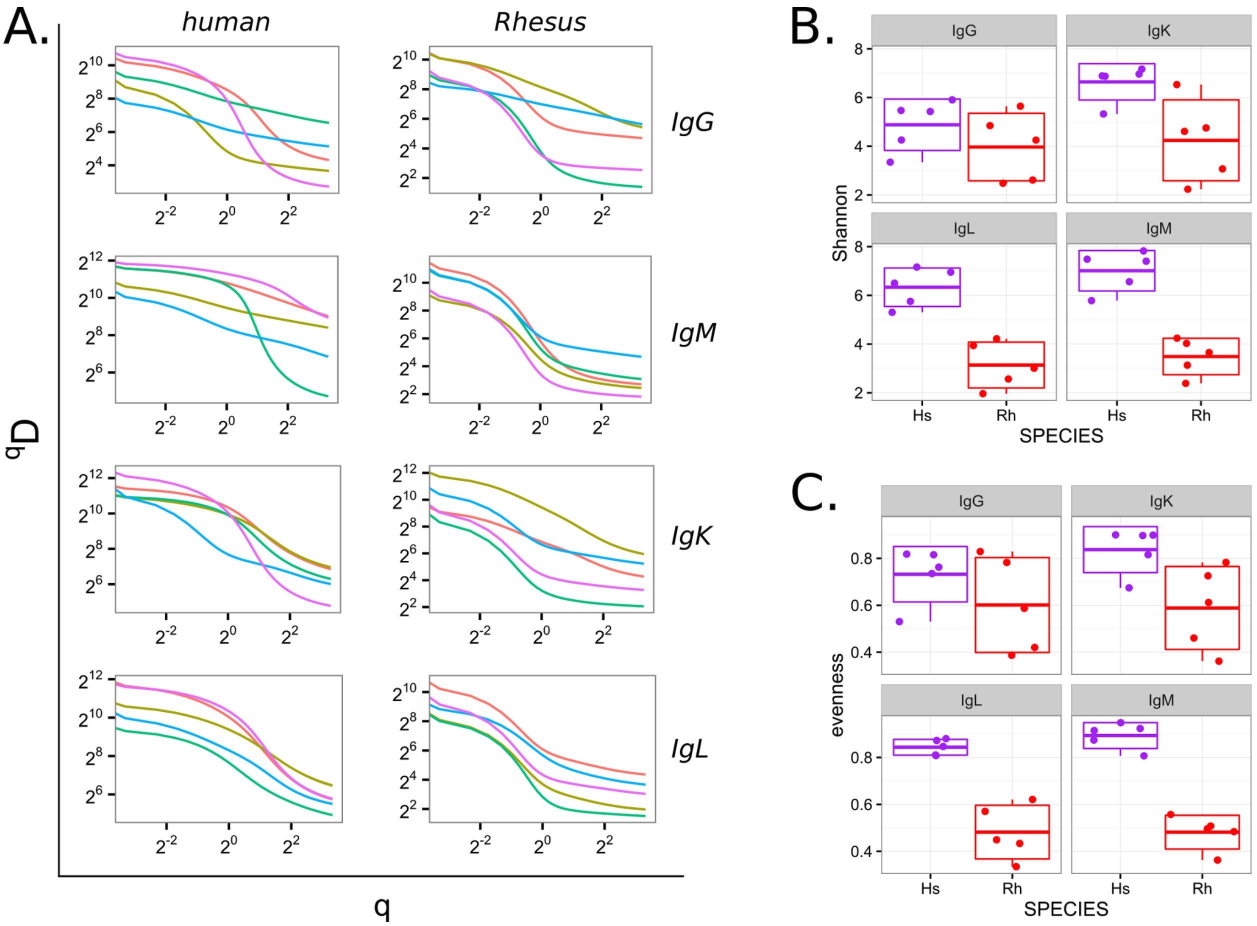

Sequence sets from each subject (or animal) are represented using Hill's diversity curves[1] (A), separated by chain (note that *D(0)* values are shown on the *y-axis*). Values for the Shannon's entropy (defined as ln[*D(1)*]) for each chain are shown in (B) with each datapoint representing a subject (or animal). Similarly, evenness values (ln[*D(1)* / ln[*D(0)*) for each chain are shown in (B) with each datapoint representing a subject (or animal).

Supplementary Figure 2. Distribution of V-family genes in the expressed BCR repertoires of healthy human subjects and untreated macaques (all unique sequences).

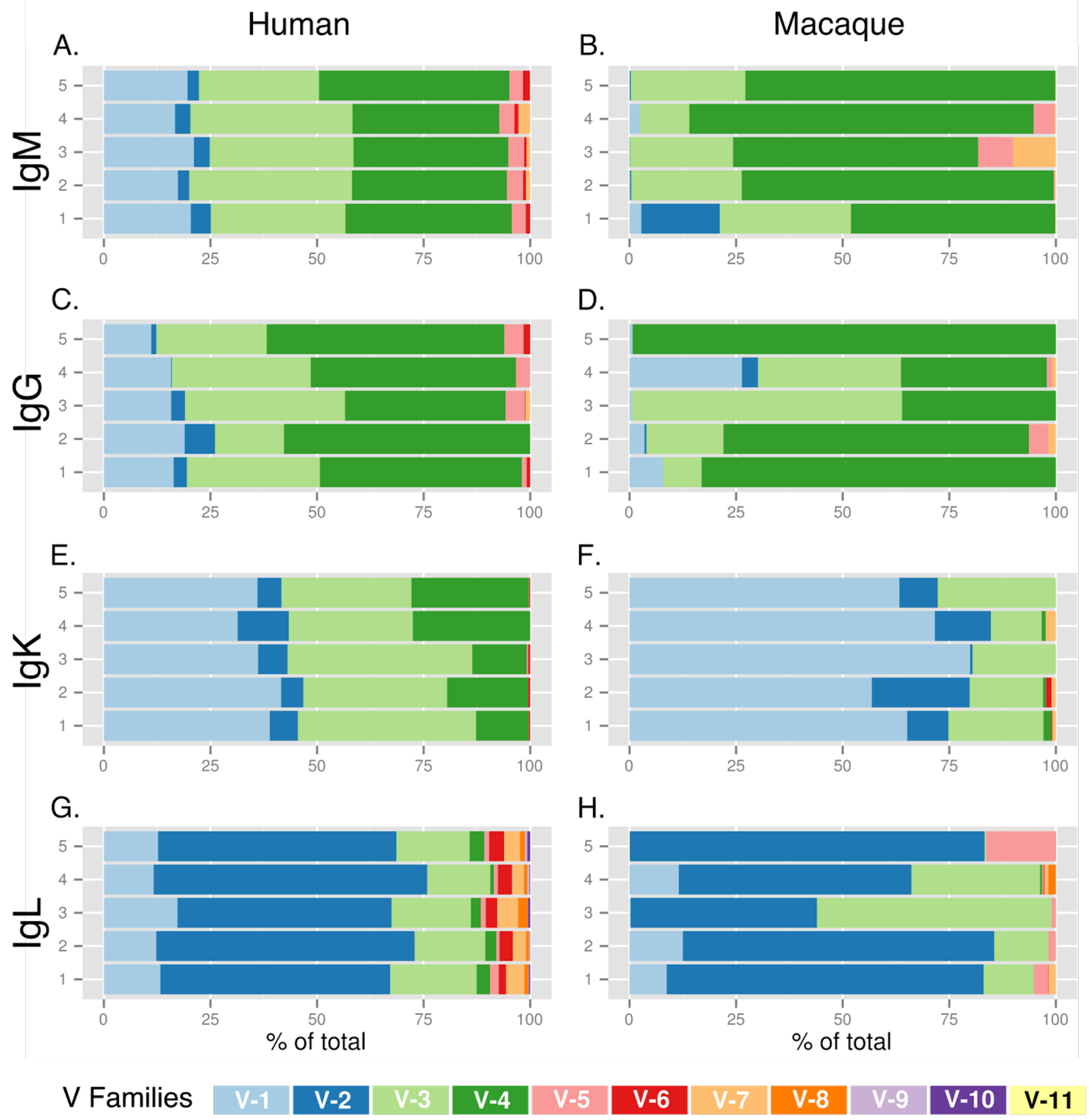

Productively rearranged sequences amplified from the expressed BCR repertoires for each human subject (*n*=5) or animal (*n*=5) are represented by horizontal bars. Each dataset consisted of >30 000 unique sequences. V-family assignments were color-coded (color assignments described below the graph) and scaled according to the proportion present in each dataset.

Supplementary Figure 3. Distribution of J-family genes in the expressed BCR repertoires of healthy human subjects and untreated macaques (all unique sequences).

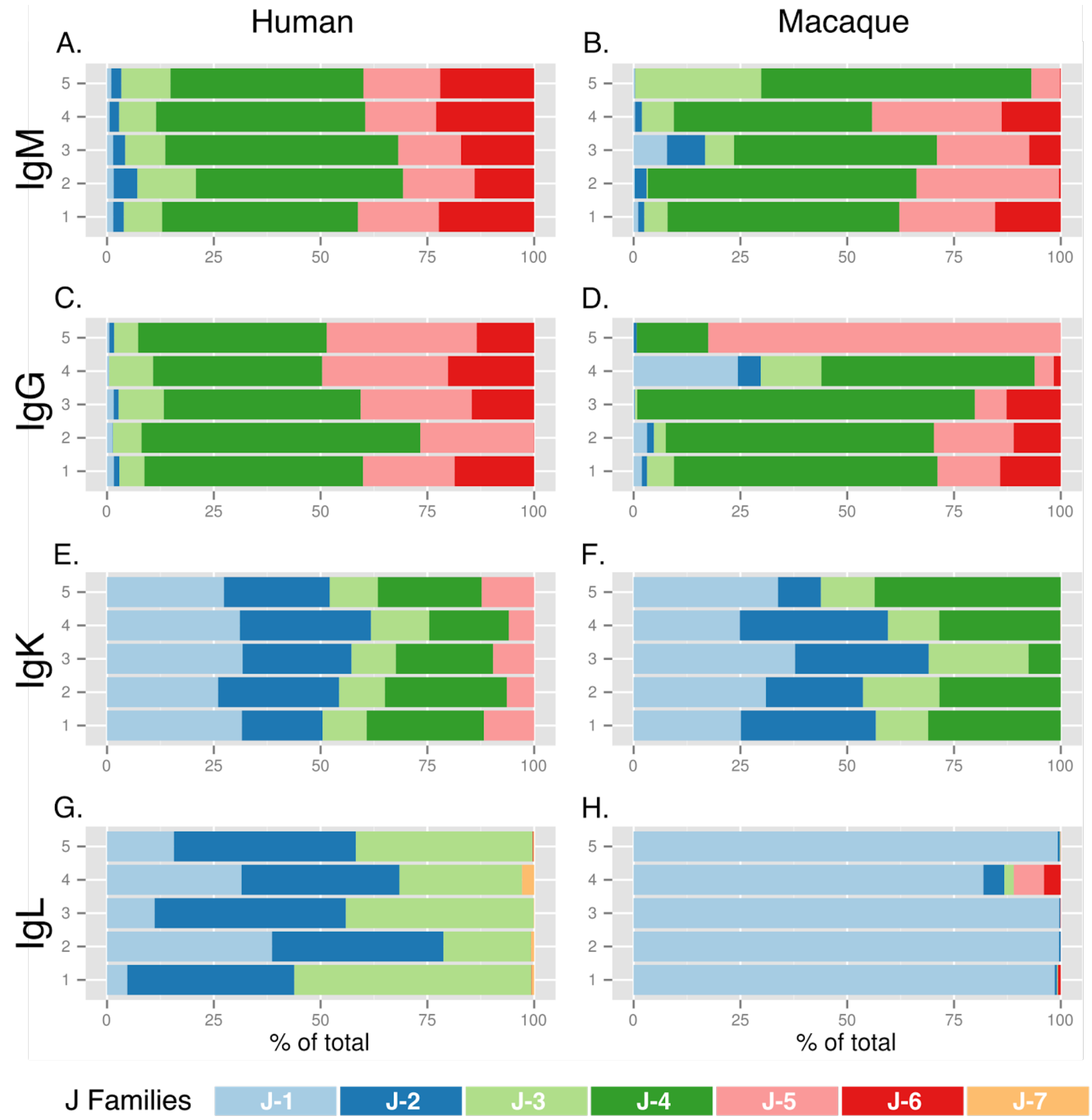

Productively rearranged sequences amplified from the expressed BCR repertoires for each human subject (*n*=5) or animal (*n*=5) are represented by horizontal bars. Each dataset consisted of >30 000 unique sequences. J-family assignments were color-coded (color assignments described below the graph) and scaled according to the proportion present in each dataset.

Supplementary Figure 4. Statistical analysis of the V gene segment family frequencies in the IgM, IgG, IgK, and IgL repertoires of humans and rhesus macaques.

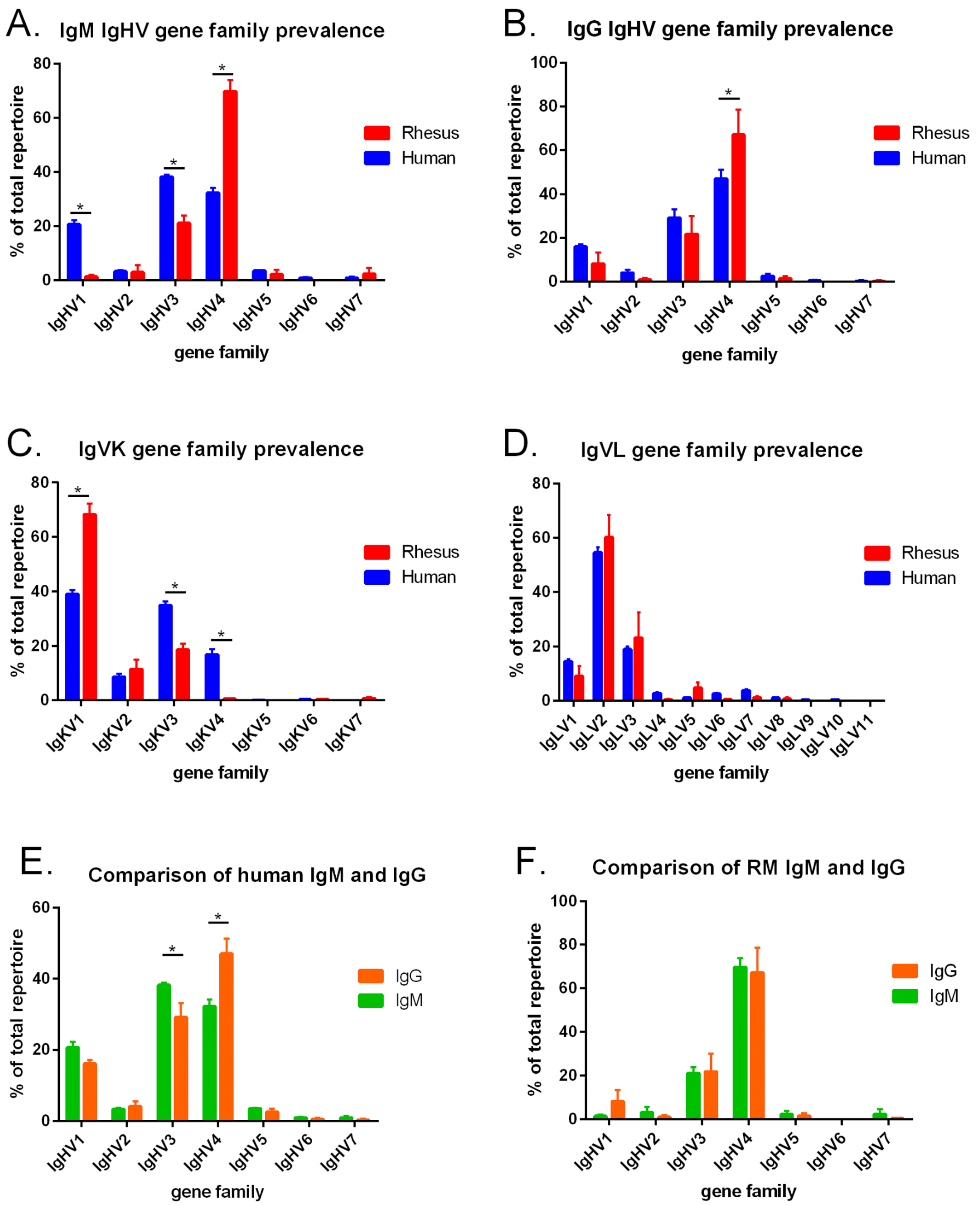

Data reported in Figures 2 were replotted as bar graphs to facilitate visual comparisons. Bars represent the mean for each data set, and the error bars reflect the standard error of mean. Statistically significant differences (P < 0.05 by Sidak's multiple comparisons test with Sidak's correction for multiple comparisons) are indicated by asterisk (*) above the data.

Supplementary Figure 5. Statistical analysis of the J gene segment family frequencies in the IgM, IgG, IgK, and IgL repertoires of humans and rhesus macaques.

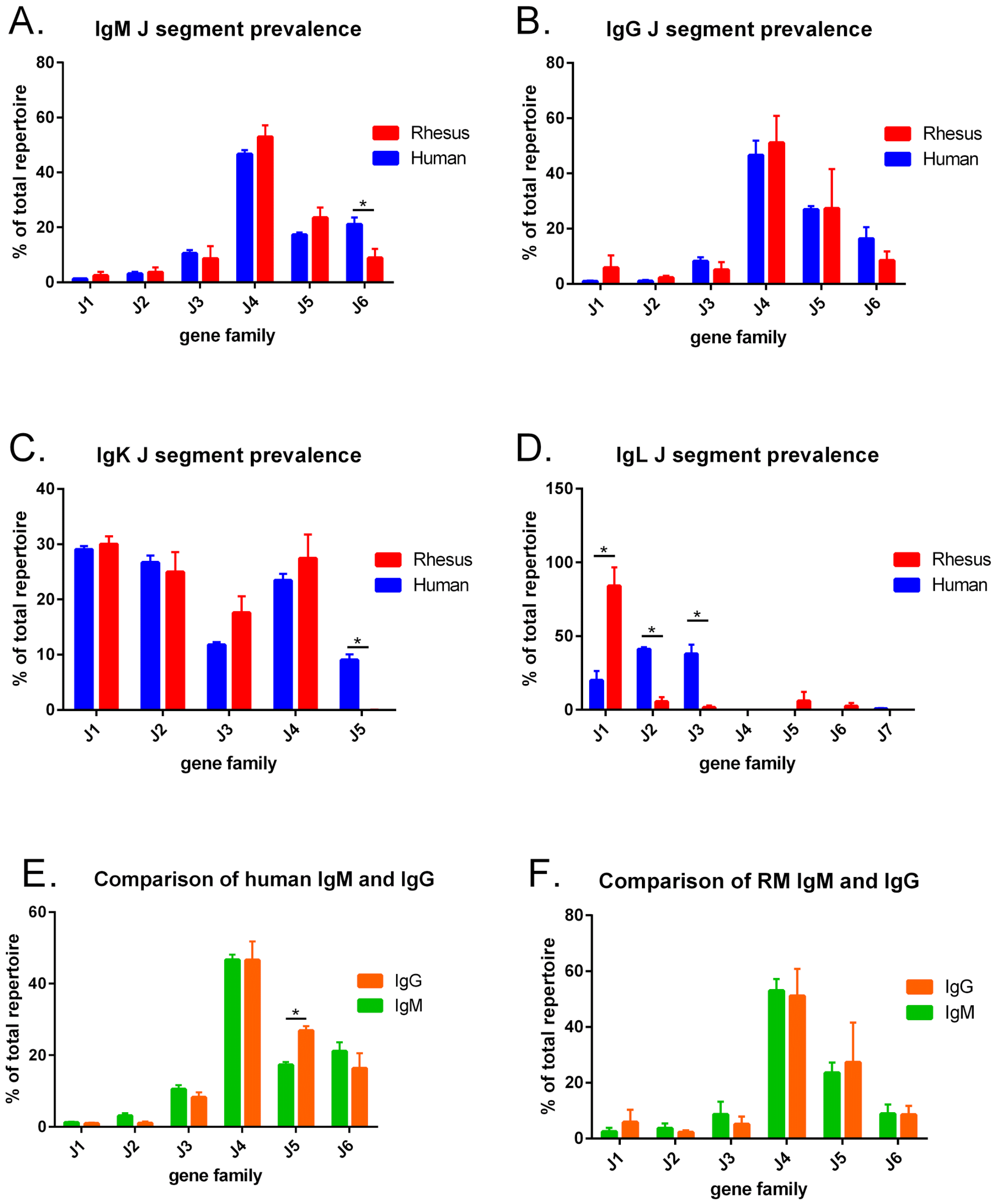

Data reported in Figure 3 were replotted as bar graphs to facilitate visual comparisons. Bars represent the mean value for each data, and the error bars reflect the standard error of mean. Statistically significant differences (P < 0.05 by Sidak's multiple comparisons test with Sidak's correction for multiple comparisons) are indicated by asterisk (*) above the data.

Supplementary Figure 6. Statistical analyses of the CDRH3 and CDRL3 lengths in IgM, IgG, IgK, and IgL repertoires of humans and rhesus macaques.

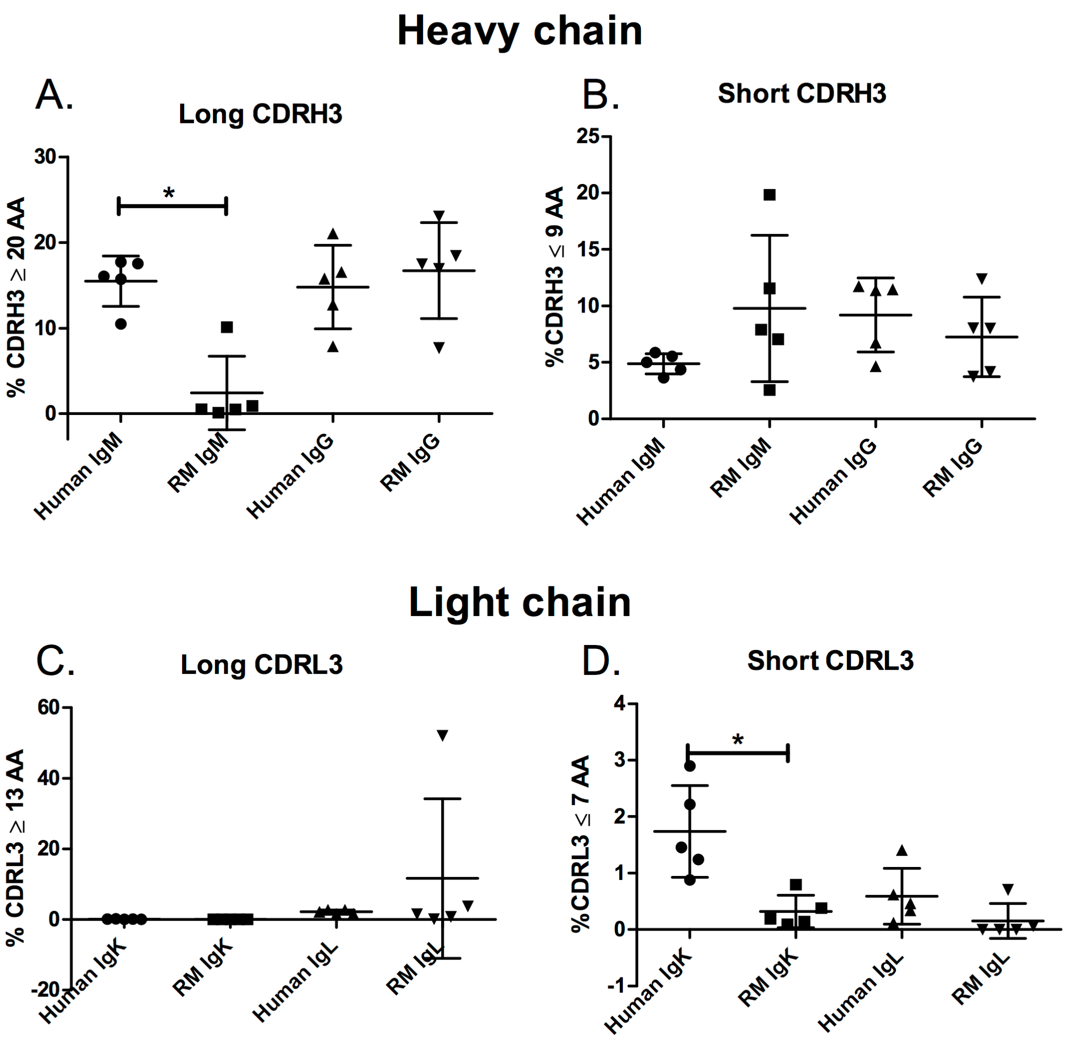

Data reported in Figures 4 were replotted to facilitate visual comparisons. Statistically significant differences (P < 0.05 by parametric unpaired t test) are indicated by asterisk (*) above the data.

Supplementary Figure 7. Frequency of IGHV1-2*02 gene in human BCR repertoires and its rhesus homolog IGHV1-KI (VH1.23) in rhesus macaque BCR repertoires.

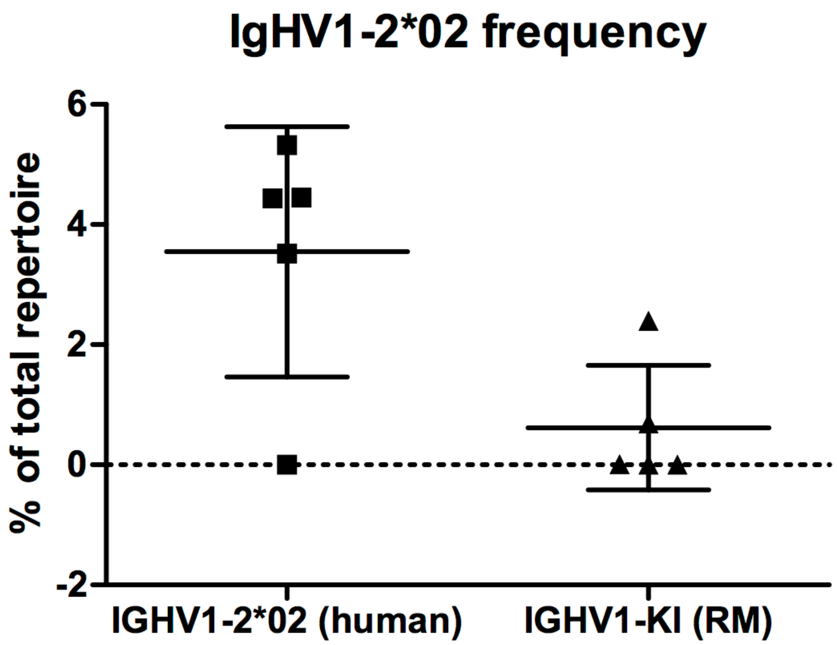

Proportion (relative to the total repertoire) of productively rearranged sequences amplified from the expressed BCR repertoires for each human subject (*n*=5) or animal (*n*=5) and annotated as IGHV1-2*02 (for human sequences) and IGHV1-KI (for macaque sequences) are represented filled squares and triangles, respectively. Each dataset consisted of >30,000 unique sequences.

Table S1. Primer sets used in the construction of Illumina sequencing libraries for rhesus macaques

A. IgM primers

3'IgM (OUTER)	CCACTTCGTTTGTATCCAACG
3'IgM (INNER)	GCATTCTCACAGGAGACGAGG
MiSeq HsRh IgM CH1 R	*GTCTCGTGGGCTCGGAGATGTGTATAAGAGACAG*GGGTTGGGGCGGATGCACT

B. IgG primers

3'IgG (OUTER)	GCCGGGAAGGTGTGCACGCCGCTGGTC
3'IgG (INNER)	CCGGTTCAGGGAAGTAGTCCTTGAC
MiSeq HsRh IgG CH1 R	*GTCTCGTGGGCTCGGAGATGTGTATAAGAGACAG*GGGGGAAGACCGATGGGCCCTT

C. IgK primers

3'IgK (OUTER)	GTCCTGCTCTGTGACACTCTC
3'IgK (INNER)	ATTCAGCAGGCACACAACAGAGGC
MiSeq HsRh IgK CL1 R	*GTCTCGTGGGCTCGGAGATGTGTATAAGAGACAG*GAAGACAGATGGTGCAGCC

D. IgL primers

3'IgL (OUTER)	TGTTGCTCTGTTTGGAGGG
3'IgL (INNER)	AGACACACTAGTGTGGCCTTG
MiSeq HsRh IgL CL1 R	*GTCTCGTGGGCTCGGAGATGTGTATAAGAGACAG*GGAACAGAGTGACCGTGGG

E. Universal forward primer

MiSeq SMARTer IIA	*TCGTCGGCAGCGTCAGATGTGTATAAGAGACAG*AAGCAGTGGTATCAACGC

Reference

1 Hill MO. Diversity and Evenness: A Unifying Notation and Its Consequences. *Ecology* 1973; **54**: 427–432.
